# Supplementary material for: The tyrosine phosphatases LAR and PTPRδ act as receptors of the nidogen-tetanus toxin complex
Source: EMBO J. 2024 Jul 8;43(16):5. doi: 10.1038/s44318-024-00164-8 (PMC11329502; doi:10.1038/s44318-024-00164-8)
Supplement: Supplementary file 1 — Appendix [file 44318_2024_164_MOESM1_ESM.pdf]

**Appendix for:**

**The tyrosine phosphatases LAR and PTPRδ act as receptors of the nidogen-tetanus toxin complex**

## Table of Contents


| Name | Page no. | Descriptor |
|---|---|---|
| Appendix Figure S1 | 2 | Neuronal PTPRσ does not interact with nidogens. |
| Appendix Figure S2 | 3 | Bacterial purification of the LAR 2nd and 4th FNIII domains. |
| Appendix Figure S3 | 4 | Bacterial purification of the LAR 5th and 7th FNIII domains. |
| Appendix Figure S4 | 5 | Purification of the LAR FNIII1-4-FLAG and FNIII5-7-FLAG fragments. |
| Appendix Figure S5 | 6 | Purification of the PTPRδ Ig1-3-His and FNIII5-7-FLAG fragments. |

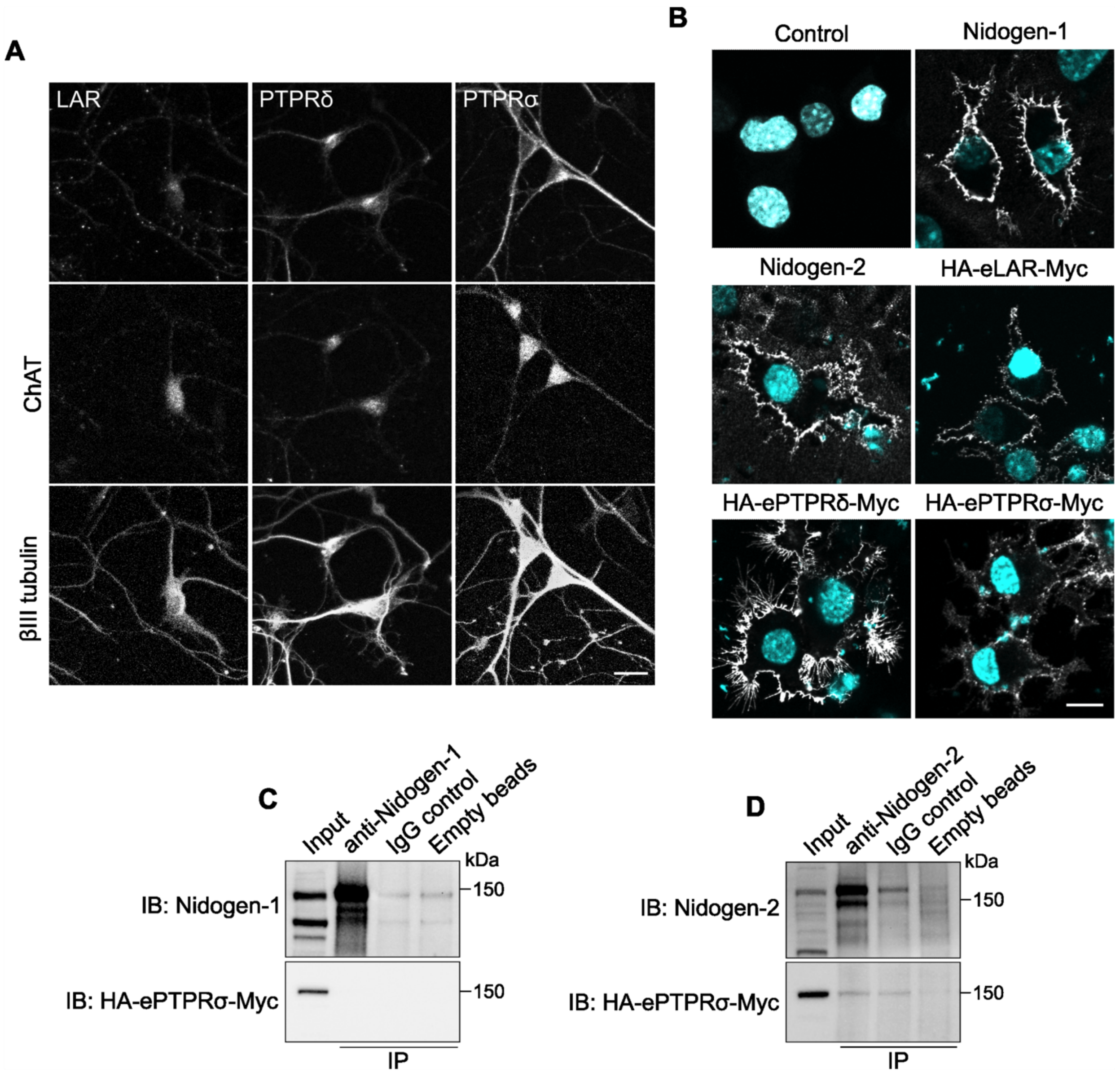


**Appendix Figure S1. Neuronal PTPRσ does not interact with nidogens.**

**A** Representative images of ventral horn cultures stained for the motor neuron marker choline acetyltransferase (ChAT), pan-neuronal marker βIII-tubulin and LAR family members. Scale bar: 20 μm. **B** Representative images showing localisation of recombinant nidogen-1, nidogen-2, eLAR, ePTPRδ and ePTPRσ in mouse N2a cells upon transient overexpression. Nuclei stained with 4′,6-diamidino-2-phenylindole (DAPI) have been pseudo-coloured in cyan. Scale bar: 5 μm.

**C, D** Unlike LAR and PTPRδ, PTPRσ does not interact with nidogens in the presence of $H_CT$. Nidogens were immunoprecipitated from N2a cell lysates, which were then probed for HA-ePTPRσ-Myc using an anti-HA antibody. Non-specific antibodies bound to beads and empty beads were used as negative controls; 5% input was loaded.

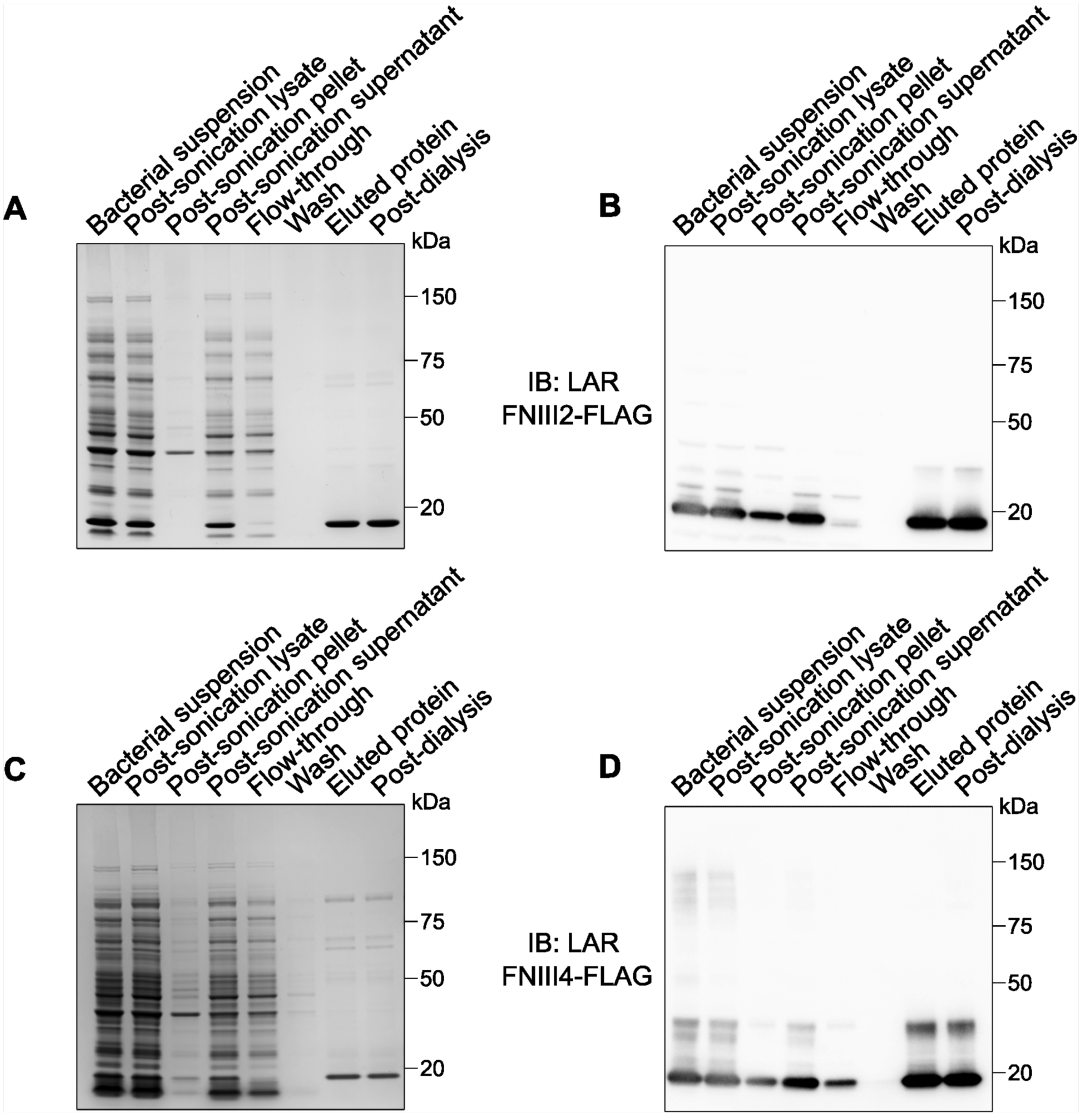


**Appendix Figure S2. Bacterial purification of the LAR 2nd and 4th FNIII domains.**

**A**, **B** Coomassie staining (A) and western blot (B) showing bacterial expression and $Ni^{2+}$-affinity purification of LAR FNIII2-FLAG (predicted molecular weight is ~15 kDa).

**C, D** Coomassie staining (C) and western blot (D) showing bacterial expression and $Ni^{2+}$-affinity purification of LAR FNIII4-FLAG (predicted molecular weight is ~15 kDa). Western blots were probed using an anti-FLAG antibody.

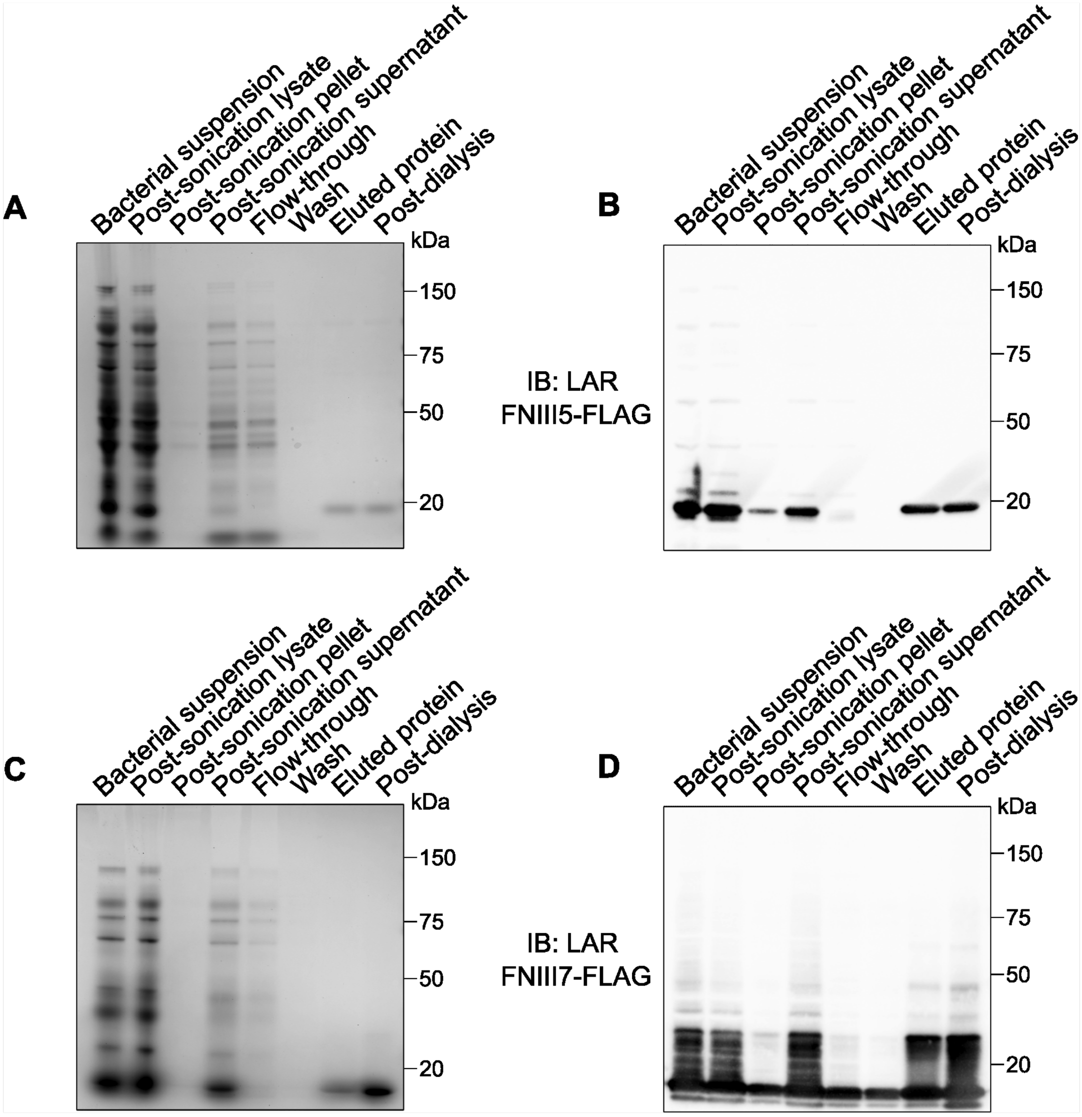


**Appendix Figure S3. Bacterial purification of the LAR 5th and 7th FNIII domains.**

**A, B** Coomassie staining (A) and western blot (B) showing bacterial expression and $Ni^{2+}$-affinity purification of LAR FNIII5-FLAG (predicted molecular weight is ~17 kDa).

**C, D** Coomassie staining (C) and western blot (D) showing bacterial expression and $Ni^{2+}$-affinity purification of LAR FNIII7-FLAG (its predicted molecular weight is ~15 kDa). Western blots were probed using an anti-FLAG antibody.

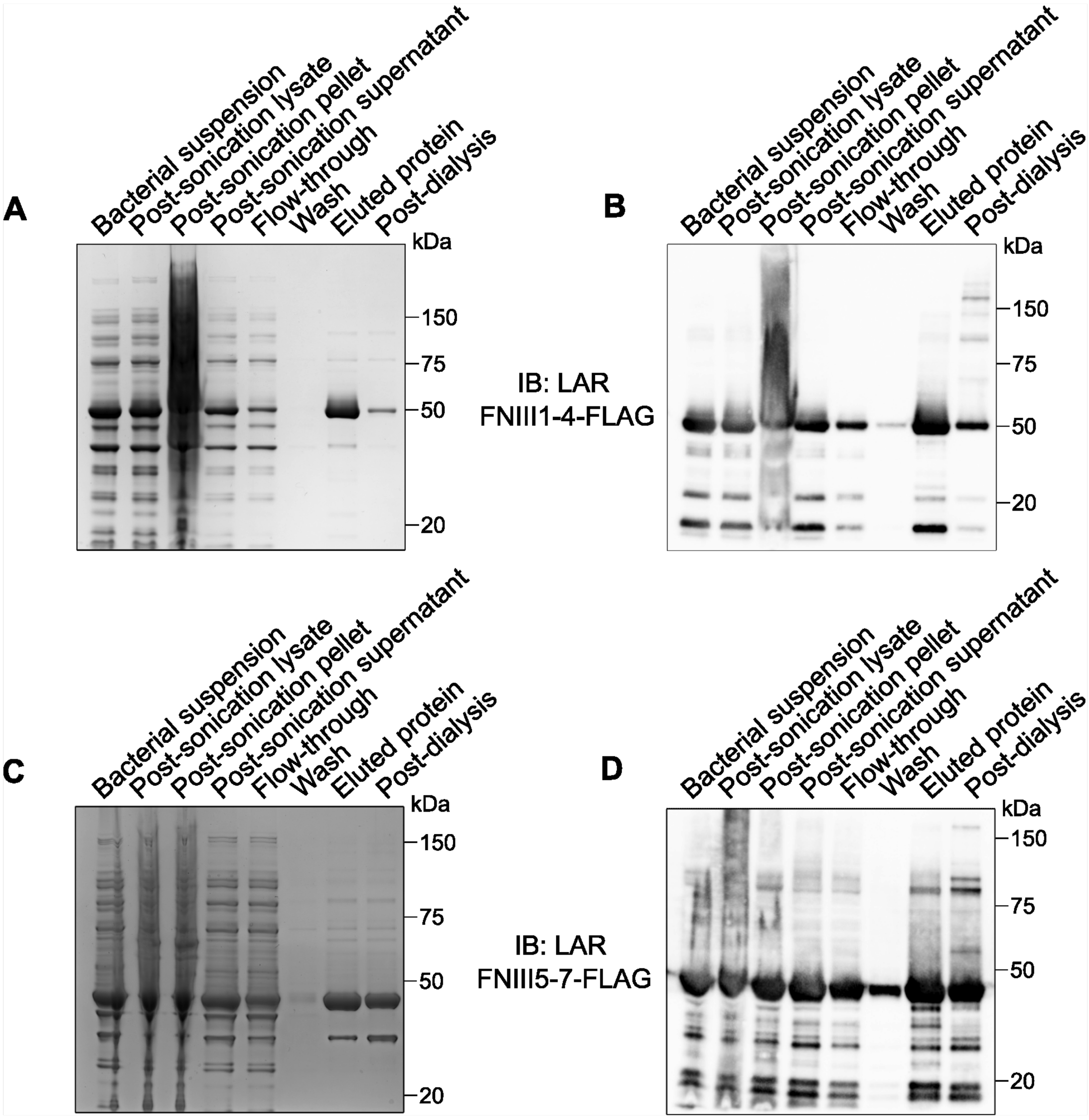


**Appendix Figure S4. Purification of the LAR FNIII1-4-FLAG and FNIII5-7-FLAG fragments.**

**A, B** Coomassie staining (A) and western blot (B) showing bacterial expression and $Ni^{2+}$-affinity purification of LAR FNIII1-4-FLAG (predicted molecular weight is ~47 kDa).

**C, D** Coomassie staining (C) and western blot (D) showing bacterial expression and $Ni^{2+}$-affinity purification of LAR FNIII5-7-FLAG (predicted molecular weight is ~39 kDa). Western blots were probed using an anti-FLAG antibody.

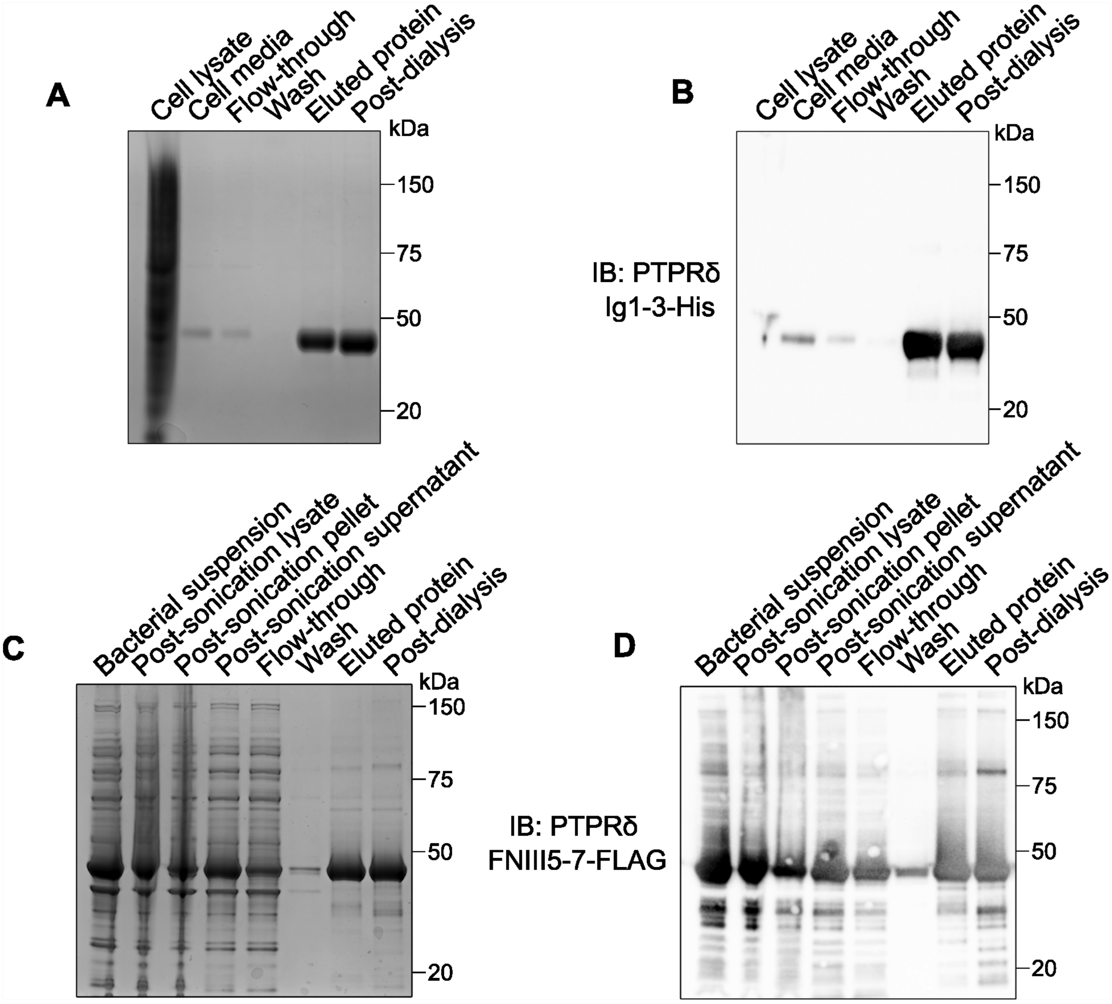


**Appendix Figure S5. Purification of the PTPRδ Ig1-3-His and FNIII5-7-FLAG fragments.**

**A, B** Coomassie staining (A) and western blot (B) showing the expression and $Ni^{2+}$-affinity purification of PTPRδ Ig1-3-His from Expi293F cells (predicted molecular weight is ~38 kDa). Western blots were probed using an anti-His antibody.

**C, D** Coomassie staining (C) and western blot (D) showing bacterial expression and $Ni^{2+}$-affinity purification of PTPRδ FNIII5-7-FLAG (predicted molecular weight is ~38 kDa). Western blots were probed with an anti-FLAG antibody.
