# Supplementary material for: The tyrosine phosphatases LAR and PTPRδ act as receptors of the nidogen-tetanus toxin complex
Source: EMBO J. 2024 Jul 8;43(16):5. doi: 10.1038/s44318-024-00164-8 (PMC11329502; doi:10.1038/s44318-024-00164-8)
Supplement: Supplementary file 4 — Movie EV2 [file 44318_2024_164_MOESM4_ESM.zip › EMBOJ-2023-113683R-Q_Movie EV2 Legend.docx]

**Movie EV2.** Mice injected with TeNT develop severe gait abnormalities at 96 h post-injection, with permanent plantar flexion of the affected hind paw.
