# Supplementary material for: The tyrosine phosphatases LAR and PTPRδ act as receptors of the nidogen-tetanus toxin complex
Source: EMBO J. 2024 Jul 8;43(16):5. doi: 10.1038/s44318-024-00164-8 (PMC11329502; doi:10.1038/s44318-024-00164-8)
Supplement: Supplementary file 6 — Movie EV4 [file 44318_2024_164_MOESM6_ESM.zip › EMBOJ-2023-113683R-Q_Movie EV4 Legend.docx]

**Movie EV4.** Mice co-administered with TeNT and the PTPRδ fragments Ig1-3-His and FNIII5-7-FLAG display a significant improvement in gait defects and posture at 96 h post-injection.
