# Supplementary material for: The tyrosine phosphatases LAR and PTPRδ act as receptors of the nidogen-tetanus toxin complex
Source: EMBO J. 2024 Jul 8;43(16):5. doi: 10.1038/s44318-024-00164-8 (PMC11329502; doi:10.1038/s44318-024-00164-8)
Supplement: Supplementary file 7 — Movie EV5 [file 44318_2024_164_MOESM7_ESM.zip › EMBOJ-2023-113683R-Q_Movie EV5 Legend.docx]

**Movie EV5.** Mice administered with TeNT and the four nidogen-binding fragments of both LAR and PTPRδ exhibit minimal gait abnormalities at 96 h post-injection.
