# Supplementary material for: The tyrosine phosphatases LAR and PTPRδ act as receptors of the nidogen-tetanus toxin complex
Source: EMBO J. 2024 Jul 8;43(16):5. doi: 10.1038/s44318-024-00164-8 (PMC11329502; doi:10.1038/s44318-024-00164-8)
Supplement: Supplementary file 8 — Expanded View Figures [file 44318_2024_164_MOESM8_ESM.pdf]

Expanded View Figures

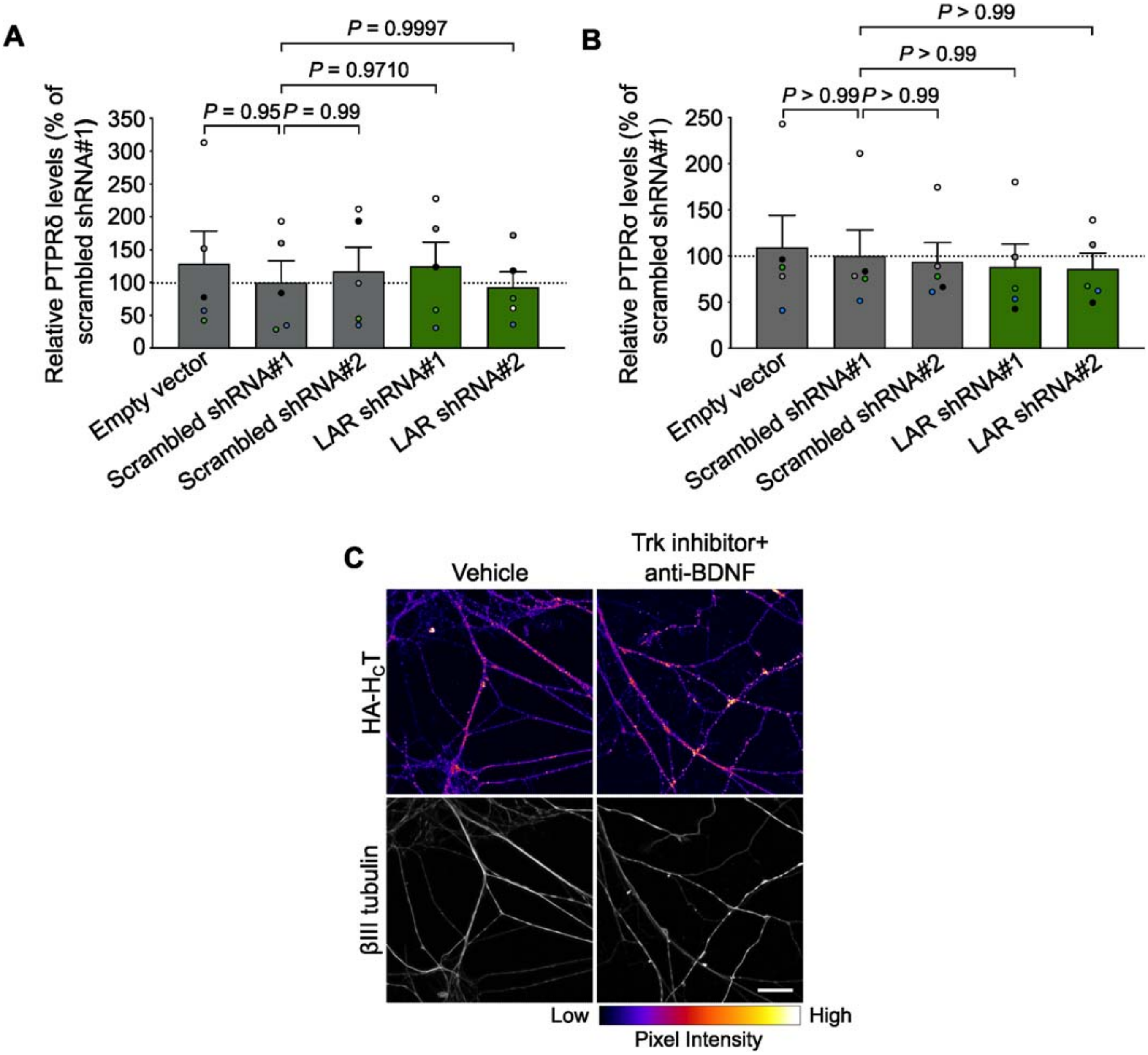

Figure EV1. Lentivirus-mediated LAR knockdown has no effect on expression levels of PTPRδ and PTPRσ.

(**A**) PTPRδ quantification in lysates of ventral horn cultures transduced with lentiviruses encoding shRNAs against mouse LAR. Results were tested for statistical significance using one-way ANOVA ($P = 0.9480$), followed by Dunnett's multiple comparisons test ($n = 5$ independent experiments; error bars indicate s.e.m.). (**B**) PTPRσ quantification in lysates of ventral horn cultures transduced with lentiviruses encoding shRNAs against mouse LAR. Results were tested for statistical significance using Kruskal–Wallis test ($P = 0.9665$), followed by Dunn's *post-hoc* test ($n = 5$ independent experiments; error bars indicate s.e.m.). Data are presented as a percentage of the total levels of PTPRδ (**A**) and PTPRσ (**B**) in neurons treated with scrambled shRNA#1. (**C**) Representative confocal images of endocytosed HA-H_CT in motor neurons treated with the pan-Trk inhibitor PF-06273340 and an anti-BDNF antibody. Images in the top panel have been colour mapped based on their intensities. Vehicle refers to DMSO-treated cultures. Scale bar: 20 µm.

© The Author(s)

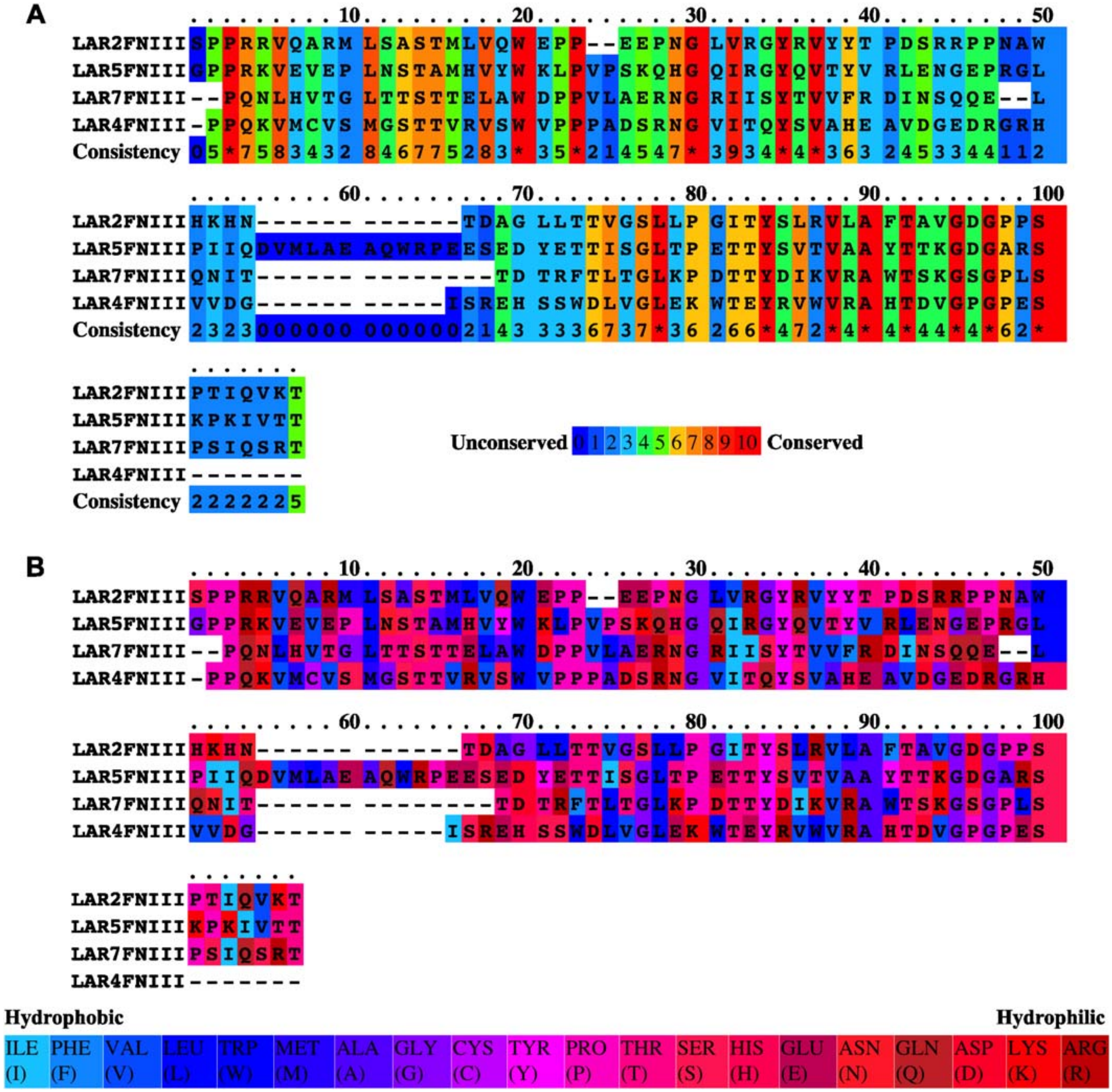

Figure EV2. Sequence alignment of the human LAR FNIII2, FNIII4, FNIII5 and FNIII7 domains using PRALINE.

(**A**) Sequence alignment of the human LAR FNIII2, FNIII4, FNIII5 and FNIII7 domains. Scores range from 0 for the least conserved alignment position, up to 10 for the most conserved position. (**B**) Conservation of hydrophobicity/hydrophilicity in the nidogen-binding domains of LAR. Colour assignments from hydrophobic to hydrophilic are shown below the alignment.

 © The Author(s)

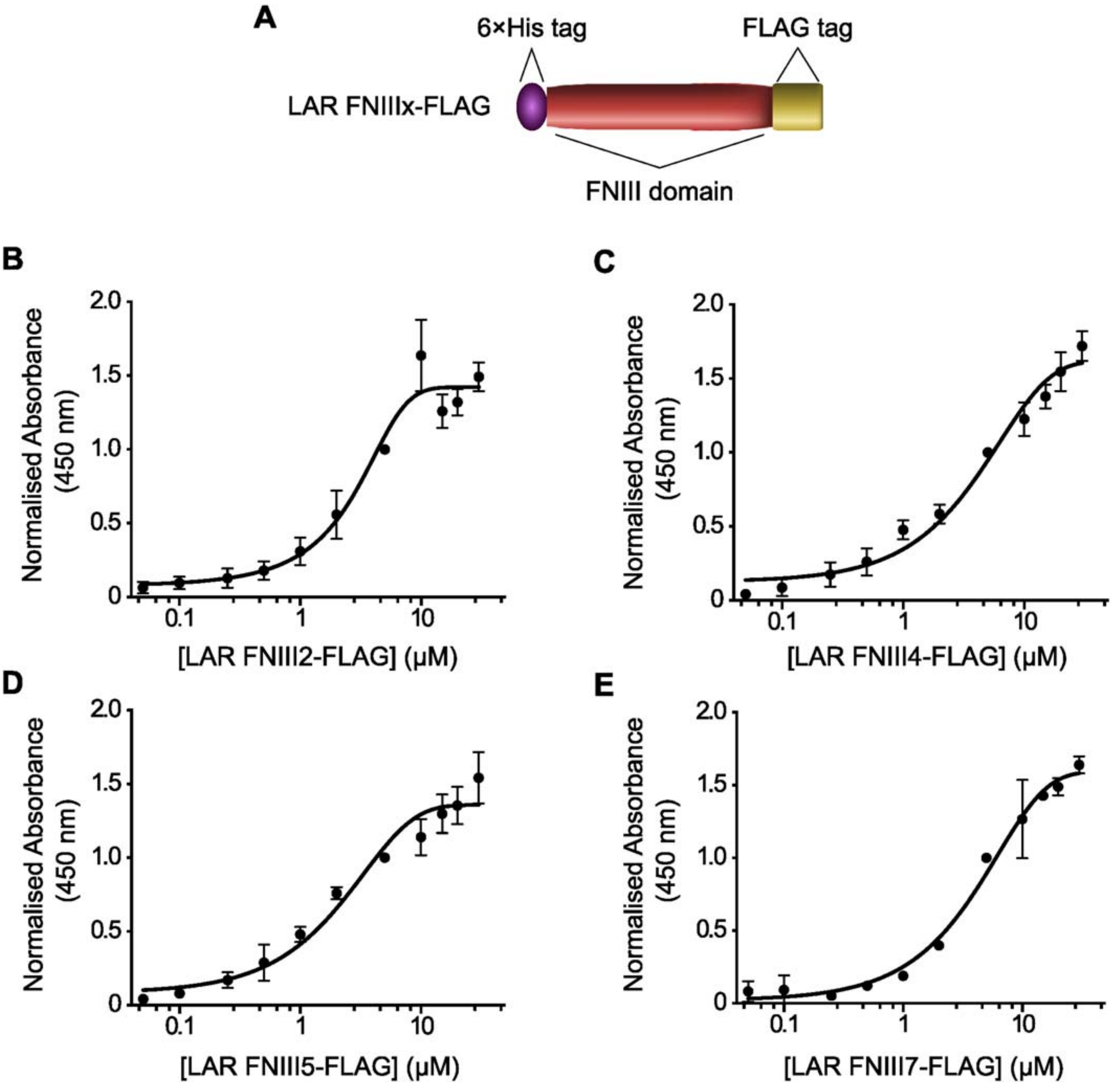

Figure EV3. Dose-dependence of the interaction between nidogen-2 and soluble LAR FNIII2, FNIII4, FNIII5 and FNIII7 domains.

(**A**) Schematic of LAR FNIII fusion proteins used for bacterial expression and purification. Each nidogen-binding FNIII domain was tagged with a 6×His tag at the N-terminus and a FLAG tag at the C-terminus. (**B–E**) Plots showing in vitro binding between purified nidogen-2 and bacterially expressed LAR FNIII2-FLAG (**B**), LAR FNIII4-FLAG (**C**), LAR FNIII5-FLAG (**D**), and LAR FNIII7-FLAG (**E**). Serial dilutions of each purified LAR FNIII domain (50 nM–30 μM) were added to a fixed amount of immobilised nidogen-2 (0.5 picomoles), followed by addition of an anti-FLAG antibody to reveal complex formation using ELISA. All datapoints were normalised to the absorbance obtained using 5 μM of LAR FNIII domain ($n = 3$ independent experiments; error bars indicate s.e.m.).

© The Author(s)

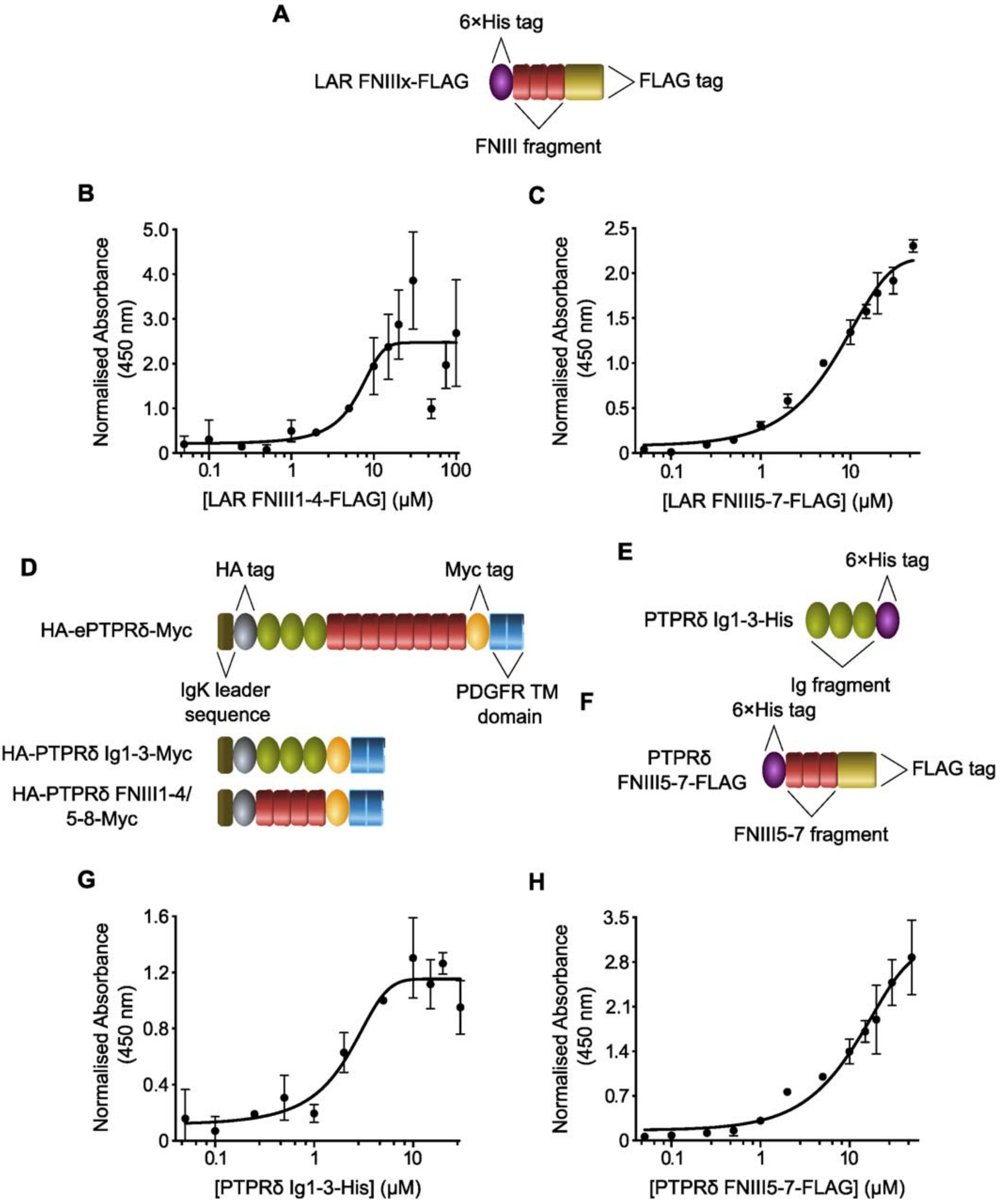
A
6×His tag
LAR FNIIIx-FLAG
FLAG tag
FNIII fragment
B
Normalised Absorbance (450 nm)
5.0
4.0
3.0
2.0
1.0
0
0.1
1
10
100
[LAR FNIII1-4-FLAG] (μM)
C
Normalised Absorbance (450 nm)
2.5
2.0
1.5
1.0
0.5
0
0.1
1
10
[LAR FNIII5-7-FLAG] (μM)
D
HA tag
Myc tag
HA-ePTPRδ-Myc
IgK leader sequence
PDGFR TM domain
HA-PTPRδ Ig1-3-Myc
HA-PTPRδ FNIII1-4/5-8-Myc
E
6×His tag
PTPRδ Ig1-3-His
Ig fragment
F
6×His tag
PTPRδ FNIII5-7-FLAG
FLAG tag
FNIII5-7 fragment
G
Normalised Absorbance (450 nm)
1.6
1.2
0.8
0.4
0
0.1
1
10
[PTPRδ Ig1-3-His] (μM)
H
Normalised Absorbance (450 nm)
3.5
2.8
2.1
1.4
0.7
0
0.1
1
10
[PTPRδ FNIII5-7-FLAG] (μM)

 © The Author(s)

Figure EV4. Nidogen-2 and LAR/PTPRδ fragments exhibit dose-dependent interactions in vitro.

(**A**) Schematic of recombinant LAR FNIII fragments used for bacterial expression and purification. LAR FNIII1-4 and FNIII5-7 fragments were tagged with a 6×His tag at the N-terminus and a FLAG tag at the C-terminus. (**B**, **C**) Plots showing in vitro binding between purified nidogen-2 and bacterially expressed LAR FNIII1-4-FLAG (**B**) and LAR FNIII5-7-FLAG (**C**). Serial dilutions of each purified LAR fragment were added to a fixed amount of immobilised nidogen-2 (0.5 picomoles), followed by addition of an anti-FLAG antibody to reveal complex formation using ELISA. All datapoints were normalised to the absorbance obtained using 5 µM of LAR FNIII fragment ($n = 3$ independent experiments; error bars indicate s.e.m.). (**D**) Schematic of PTPRδ fragments used to identify the interacting domains between PTPRδ and nidogens. Truncated proteins were fused to the murine Igκ-chain leader sequence and an HA tag at the N-terminus; the C-terminus was fused to the PDGFR transmembrane domain and a Myc tag. (**E**, **F**) Schematic of recombinant PTPRδ fragments used for protein expression and purification. PTPRδ Ig1-3 was tagged with a 6×His tag at the C-terminus (**E**), while the FNIII5-7 fragment was tagged with a 6×His tag at the N-terminus and a FLAG tag at the C-terminus (**F**). (**G, H**) Plots showing in vitro binding between purified nidogen-2 and recombinant PTPRδ Ig1-3-His (**G**) and PTPRδ FNIII5-7-FLAG (**H**). Serial dilutions of each PTPRδ fragment were added to a fixed amount of immobilised nidogen-2 (0.5 picomoles), followed by addition of an anti-His or anti-FLAG antibody, respectively, to reveal complex formation using ELISA. All datapoints were normalised to the absorbance obtained using 5 µM of PTPRδ FNIII fragment ($n = 3$ independent experiments; error bars indicate s.e.m.).

© The Author(s)

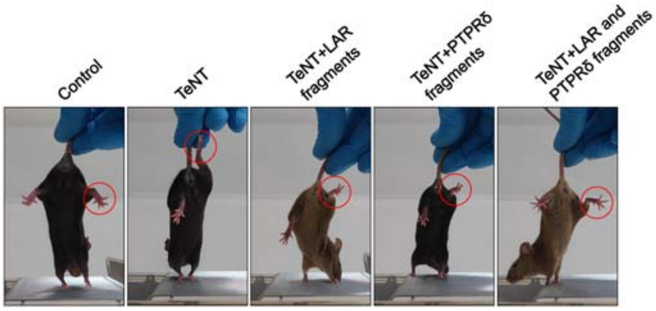

Figure EV5. Soluble LAR and PTPRδ fragments rescue posture defects in TeNT-injected mice.

Representative still images showing the outcomes of a tail suspension assay in mice injected with sub-lethal doses of TeNT alone, or TeNT pre-mixed with LAR and/or PTPRδ fragments. Mice were injected in the gastrocnemius muscle of the right hindlimb (red circles); non-injected mice were used as a negative control.

 © The Author(s)
